# The relationship between level of knowledge and vaginal discharge prevention behavior for nursing student

**DOI:** 10.1590/0034-7167-2022-0602

**Published:** 2023-12-08

**Authors:** Antonia Diti Juniar, Arusta Yohana Simamora, Corry Natasya Parniaty Manalu, Joice Cathryne, Mega Tri Anggraini Setia Ningsih

**Affiliations:** I Universitas Pelita Harapan. Tangerang, Banten, Indonesia

**Keywords:** Vaginal Discharge, Knowledge, Behavior, Students, Nursing, Descarga Vaginal, Conhecimento, Comportamento, Estudantes, Enfermagem, Excreción Vaginal, Conocimiento, Conducta, Estudiantes, Enfermería

## Abstract

**Objective:**

Reproductive health in adolescent girls is very important. To determine the relationship between the level of knowledge and the behavior of preventing vaginal discharge among nursing students.

**Methods:**

a quantitative, cross-sectional study, with 155 first-year female students at a private faculty of nursing. Data were collected from February to March 2022, using an electronic questionnaire.

**Results:**

98.1% of participants have a good level of knowledge and 92.3% of participants practice good vaginal discharge prevention behavior.

**Conclusion:**

good knowledge produces appropriate prevention behavior. The result of this study can be used as a contribution of thoughts and references as a more in-depth study of the factors that influence the level of knowledge and behavior about the disease of the genitalia and the dangers of pathological vaginal discharge.

## RESUMO

**Objetivo:**

a saúde reprodutiva das adolescentes é muito importante. Determinar a relação entre o nível de conhecimento e o comportamento de prevenção do corrimento vaginal entre estudantes de enfermagem.

**Método:**

estudo quantitativo, transversal, com 155 estudantes do primeiro ano de uma faculdade privada de enfermagem. Os dados foram coletados entre fevereiro e março de 2022 por meio de questionário eletrônico.

**Resultados:**

participantes com bom nível de conhecimento corresponderam a 98,1%, e as que praticam bons comportamentos de prevenção do corrimento vaginal representaram 92,3%.

**Conclusão:**

um bom conhecimento produz um comportamento de prevenção adequado. O resultado deste estudo pode contribuir com reflexões e referências para um estudo mais aprofundado dos fatores que influenciam o nível de conhecimento e comportamento sobre a doença da genitália e os perigos do corrimento vaginal patológico.

## RESUMEN

**Objetivo:**

la salud reproductiva de las adolescentes es muy importante. Determinar la relación entre el nivel de conocimientos y la conducta de prevención del flujo vaginal en estudiantes de enfermería.

**Métodos:**

estudio cuantitativo, transversal, con 155 estudiantes de primer año de una facultad privada de enfermería. Los datos se recopilaron de febrero a marzo de 2022 a través de un cuestionario electrónico.

**Resultados:**

los participantes con buen nivel de conocimientos correspondieron al 98,1% y los que practican buenas conductas para prevenir el flujo vaginal, el 92,3%.

**Conclusión:**

un buen conocimiento produce conductas de prevención adecuadas. El resultado de este estudio puede contribuir con reflexiones y referencias para un estudio más profundo de los factores que influyen en el nivel de conocimiento y comportamiento sobre la enfermedad de los genitales y los peligros del flujo vaginal patológico.

## INTRODUCTION

Reproductive health has become a government concern and is a serious problem throughout life. For all adolescents and their families to have responsible behavior is the target of reproductive health programs in Indonesia. As part of their reproductive rights, the government has supported the provision of information, counseling, and services on reproductive health as widely as possible^(1)^.

Not just being free from disease or disability in matters relating to the reproductive system, its functions, and processes, reproductive health is a state of complete physical, mental, and social well-being^(2)^. If reproductive health is not maintained properly, it can lead to serious problems, especially in adolescents. Adolescents have unique characteristics, want to imitate something they see, are often inconsistent in their behavior, and often feel anxious about changes that occur in their bodies, which can lead to serious problems if not handled properly^(3)^.

Lack of information and knowledge about changes in the reproductive system in adolescence causes shame and anxiety because it is different from their peers; this results in various problems related to their reproductive organs. One of them is the appearance of vaginal discharge in adolescent girls^(4)^.

Vaginal discharge is the discharge of abnormal secretions from the female vaginal canal, which can be physiological or pathological. Physiological vaginal discharge occurs when a woman is aroused during intercourse and during a woman’s fertile period, while pathological vaginal discharge, or what is known as abnormal vaginal discharge, occurs due to an infectious process^(3)^, which is caused by bacteria, fungi, parasites and viruses. Physiological vaginal discharge can be caused by various causes, such as the entry of foreign objects into the vagina, the use of tight pants and polyester fabrics, not regularly changing underwear or sanitary pads during menstruation and inappropriate vaginal care during menstruation^(5)^. This can happen because of the lack of knowledge of women about vaginal discharge^(1)^, whereby, if they cannot maintain their reproductive health properly, it will increase the risk of cervical cancer which can lead to death^(6)^. Cervical cancer is the leading cause of death for women in the world with recorded incidence cases reaching 19.3 million cases in 2020^(7)^.

According to the WHO, 75% of women around the world have experienced vaginal discharge once, 45% of women have experienced vaginal discharge twice, and 90% of women in Indonesia have the potential to experience vaginal discharge due to Indonesia being tropical country, so fungi can easily grow which can cause yeast infections, resulting in many cases of vaginal discharge^(8)^. According to the Centers for Disease Control and Prevention (CDC) 2020, prevalence of bacterial vaginosis in the United States in women aged 14-49 years is estimated to reach 21.2 million people or 29.2%^(9)^. Pathological vaginal discharge can also be found in Pelvic cases of Inflammatory Disesase (PID) and can also be found in the case of women who suffer from Gonorrhea^(10)^. Research which was carried out in 2018 says that in Asia there are women’s reproductive health problems 76% had vaginal discharge^(11)^. Vaginal discharge symptoms also occur in unmarried women or young women aged 15-24 years, approximately 31.8%, suggesting that adolescents are at higher risk of vaginal discharge^(12)^.

Based on research, the number of women who experience vaginal discharge per year continues to increase, starting from 2010 there was an increase of 52%, in 2011, there was an increase in the incidence of vaginal discharge by 60%, in 2012 there was an increase in the number of vaginal discharge by 75% and in the months January to August 2013 there was also an increase of around 75%^(13)^. Unhealthy habits of adolescent girls can cause vaginal discharge, habits such as not drying the genital area by using a dry tissue or towel, the habit of not cleaning the genitalia from front to back^(14)^ and the act of having sexual intercourse. Lack of knowledge and information about the cleanliness of the genitals will certainly have an impact on the behavior of adolescents in maintaining the cleanliness of their genitals^(15)^.

In Adawiyah’s research, in South Tangerang, it was found that 77.9% of female students had poor personal hygiene knowledge and 53% of female students experienced pathological vaginal discharge and, based on data from the Ministry of Health of the Republic of Indonesia, knowledge about reproductive health was not sufficient; this was proven by only 35.3% of female adolescents having good knowledge about reproductive health^(16)^.

In Indonesia, there are 45.3 million adolescents aged 15-24 years who have unhealthy behavior and 83.3% of 30 million adolescents aged 15-24 years have had sexual intercourse, so this is a factor causing vaginal discharge in women^(17)^. Vaginal discharge is a common problem for most women. However, at the same time, most women are not knowledgeable on vaginal discharge and what causes vaginal discharge. If not treated properly, vaginal discharge can have fatal outcomes such as resulting in infertility and ectopic^(18)^. Maintaining the cleanliness of female vital organs is very important and must be known by the public, although sometimes they have sufficient knowledge but their behavior in maintaining reproductive hygiene is often questioned^(19)^.

Based on a survey of initial data conducted on first-year nursing students at a private university in Tangerang, 60.4% of female students were experiencing vaginal discharge. Based on the above phenomenon, the researcher is interested in conducting research on “The Relationship of Knowledge Level with Vaginal Prevention Behavior in Nursing Students at a private university in Tangerang”.

## OBJECTIVE

To analyze the relationship between level of knowledge and vaginal discharge prevention behavior for nursing students.

## METHODS

### Ethical Aspect

This study complied with the ethical aspects involved in research with human beings, according to the recommendations of KEP FoN No. 039/KEPFON/I/ 2022 and was approved by Research Ethics Committee of Faculty Nursing. All participants agreed with the inform consent form available online, in which they clicked in option “I accept to participated in the study”.

### Design, period, and location

This is a descriptive cross-sectional study based on the recommendations of Enhanced Reporting of Observational Studies in Epidemiology (STROBE). The participants were first-year nursing students at a private university in Tangerang, Indonesia. Data collection took place from February 2022 to March.

### Population, inclusion and exclusion criteria

Of the 265 first-year female nursing students, 135 agreed to participate in the study, and the sample was chosen by convenience. The criteria for inclusion were a first-year female nursing student and answered the entire questionnaire, and first-year female nursing students who experiencing pathological vaginal discharge and on treatment were excluded from the study.

### Study protocol

Data collection took place between February and March 2022, exclusively online through internet access and using google form. The authors created a google forms engagement page with the objective of disseminating scientific information of instrument to obtain health information regarding knowledge and questionnaire about vaginal discharge prevention behavior.

Data collection occurred through the application of two instruments: a questionnaire prepared by the researcher themselves and questionnaire about knowledge and vaginal discharge prevention behavior. The first contemplated the characteristics of respondents, covering the variables: age, previous school, source of information, ethnic and types of vaginal discharges.

The second questionnaire, researchers adapted from Saloma^(20)^ questionnaire which had been tested for validity and reliability. This questionnaire about vaginal discharge knowledge distributed in question about definitions, sign and symptoms, causes, risk factors, the transmission, complication and treatment methods about vaginal discharge, whose assessment is measured using the Guttman scale. The questionnaire about vaginal discharge behavior which distributed in statements maintaining vaginal hygiene, how to dress normally, reducing to use of pantyliner, treatment of pathological vaginal discharge and prevention of sexual behavior risky which answered with yes or no.

### Analysis of Results and Statistics

Researchers use two types of data analysis, namely univariate and bivariate analysis. Univariate was used to determine the level of knowledge and vaginal discharge prevention behavior, the results of which were issued in the form of frequency and percentage and then grouped into three levels of knowledge, namely good, fair and poor knowledge which were divided according to predetermined ranges, as well as analysis on vaginal discharge prevention behavior, analyzed using SPSS where the results of the analysis are divided into three predetermined levels in the form of frequency and percentage. Analysis to determine the relationship between the two variables’ level of knowledge and vaginal discharge prevention behavior in which research used bivariate analysis using the Spearman Rank coefficient test.

## RESULTS

Table 1 shows the distribution of data based on the characteristics of respondents. There was a higher prevalence of age, 58.4% (n=92) of the participants were aged 18 years old who 94.8% (n=147) of participants graduated from non-health senior high school graduates, and majority 58.4% (n=92) of participants received information about vaginal discharge from the internet, all the participant coming from different ethnicity, where 24.5% (n=38) of the participants came from the Batak ethnic, and 100% (N=155) of the participants experienced with physiological vaginal discharge.

**Table 1:** Distribution of characteristics of respondents

Category	n	%
Age		
17 years old	12	7.7
18 years old	92	58.4
19 years old	43	27.7
20 years old	8	5.2
Previous school		
Health senior high school	8	5.2
Non-health senior high school	147	94.8
Sources of information		
Parents	23	14.8
School	36	23.2
Television	3	1.9
Internet	92	58.4
Newspapers/Magazines	1	0.6
Poster	0	0
Ethnic		
Batak	38	24.5
Nias	36	23.3
Alor	1	0.6
Chinese	1	0.6
Toraja	9	5.8
Kei	1	0.6
Flores	2	1.3
Mentawai	1	0.6
Semau	1	0.6
Dayak	12	7.7
Timor	7	4.5
Java	17	11
Rote	5	3.2
Sumba	1	0.6
Sabu	4	2.6
Minahasa	3	1.9
Sunda	1	0.6
Mangondow	0	0.0
Manggarai	1	0.6
Ambon	10	6.5
Sea sea	2	1.3
Bali	2	1.3
Types of Vaginal Discharge		
Physiological	155	100
Pathological	0	0

Table 2 shows the result of the level of knowledge of participants, where participants answered the knowledge questions by answering the right and wrong questions. The majority, 98.1% of the participants had a good level of knowledge about vaginal discharge (n=152) and only 1.9% (n=3) participants had a fair level of knowledge.

**Table 2:** Level of knowledge about vaginal discharge

Category	n	%
Good	152	98.1
Fair	3	1.9
Poor	0	0

Table 3 shows the distribution items of knowledge regarding vaginal discharge, it was found that only 16.1% of the participants answered correctly about complications of vaginal discharge, and only partially 59.3% of participants answered correctly about transmission of vaginal discharge.

**Table 3:** Frequency distribution of knowledge regarding vaginal discharge

Items	Correct %	Incorrect %
Definition of vaginal discharge	85.5	14.5
Characteristics of physiological vaginal discharge and pathological	68.4	31.6
Cause of vaginal discharge	68.5	31.5
Risk factor	66.7	29.3
Transmission of vaginal discharge	59.3	40.7
Vaginal complications	16.1	83.9
Treatment	92.9	7.1

Table 4 shows the result of the level of vaginal discharge prevention behavior. The majority, 92.3% (n=143) of the participants had a good level of vaginal discharge prevention behavior and only 7.7% (n=12) participants had a fair level of knowledge.

**Table 4:** Level of vaginal discharge prevention behavior

Category	n	%
Good	143	92.3
Fair	12	7.7
Poor	0	0

Table 5 show the distribution items of vaginal discharge prevention behavior, it was found that only 50.1% of participants answered correctly about how to dress normally to prevent vaginal discharge and only 63.7% of participants answered correctly about how to maintain vaginal hygiene.

**Table 5:** Frequency distribution of vaginal discharge prevention behavior

Items	Correct %	Incorrect %
Vaginal hygiene	63.7	36.3
How to dress normally	50.1	49.9
Reducing the use of pantyliner	83.9	16.1
Avoid self-medication	87.1	12.9
Prevention	98.7	1.3

Table 6 shows that 92.3% (n=143) of the participants had a good level of knowledge about vaginal discharge with good vaginal discharge prevention behavior and 5.8% (n=9) of participants with fair level vaginal discharge prevention behavior. The percentage of participants with fair level of knowledge about vaginal discharge with fair level vaginal discharge prevention behavior are 1.9% (n=3) of participants. There is relationship between level of knowledge and vaginal discharge prevention behavior *p* = 0.023 and this means the results are significant.

**Table 6:** The relationship of knowledge with vaginal discharge prevention behavior

Knowledge level	Vaginal Discharge Prevention Behavior level						Total		p value
	Good		Fair		Poor				
	n	%	n	%	n	%	n	%	
Good	143	92.3	9	5.8	0	0.0	152	98.1	0.023
Fair	0	0.0	3	1.9	0	0.0	3	1.9	
Poor	0	0.0	0	0.0	0	0.0	0	0.0	
Total	143	92.3	12	7.7	0	0.0	155	100	

## DISCUSSION

Based on the results of statistical tests that have been carried out, there is a relationship between knowledge and behavior to prevent vaginal discharge, p = 0.023. The age range of a woman experiencing vaginal discharge is 16-24 years^(6)^. In our study, the highest prevalence of participants had age 18 years old, which is the normal range for vaginal discharge, the findings of the current study are in contrast with the study conducted by Deniati^(21)^ which stated that adolescents aged 16-19 years old experienced the most vaginal discharge, adolescents are more at risk of experiencing vaginal discharge that is caused by infectious and non-infectious factors. An early symptom of most reproductive system disorders is abnormal vaginal discharge, which may be physiological or pathological^(22)^. Pathological causes of vaginal discharge include genital tract malignancies, fistulae, allergic reactions, menopause, and genital tract infection^(23)^. The most common causes of vaginal discharge are physiological causes, bacterial vaginosis, and candidiasis^(24)^. Based on the table 1 it was found that 100% of participants experienced physiological vaginal discharge, if vaginal discharge is not treated properly it will result in infertility^(25)^.

Educational background can add insight to one’ knowledge which can also significantly increase awareness of one’ healthy attitudes and behavior^(15)^. The results showed that 98.4% of respondents were graduates from non-health senior high schools. According to a study by Ebrahimi et al, “counseling and education for effective preventive health behaviors is one of the hottest topics in sexuality and reproductive health”. Health education should target young women to help detect abnormal vaginal discharge as early as possible^(26)^. Table 3 shows that most of the participants 83.9% answered incorrectly regarding the complications from vaginal discharge. According to research conducted by Ilankoon et al, women’s education seems to be an important factor that helps prevent reproductive complications and health disease^(27)^. Vaginal discharge complications can lead to infertility, ectopic pregnancy, and chronic pelvic pain inflammation^(28)^.

Social media is one health information campaign promoted in the mass community. Social media provides information about health, especially about vaginal discharge^(29)^ as the results of this study stated that 58.45% of respondents got information about vaginal discharge from the internet. The results of this study are supported by Susiloningtyas’ research, that 60% of adolescents get vaginal discharge information from the internet^(30)^, as well as in Febrary’s research, 86.2% of respondents get information about vaginal discharge from the internet^(31)^. Our research also shows that the internet, school, and parents are the most common channels that participants are looking for. The finding is like previous studies showing that the internet is the most popular source of reproductive health information^(28)^. The ease of access and sharing of the internet has improved the way people search for health information^(32)^. Furthermore, the proportion of people using internet for social networks to find health information has also increased dramatically because they can receive information immediately and act on it^(33)^. Previous research has shown that adolescents typically use the internet to personally search for sexual health issues and to gather information on health topics^(34)^. However, not all information on the internet has been researched by medical professionals, so misinformation can be misleading and cause health hazards. Another study, in Vestine’s research, 54% of respondents received vaginal discharge information from television, this is because television is in great demand as a medium of entertainment and seeking information and the parents of participants also do not allow their child to often access the internet through cellphones^(35)^. Research by Abdelnaem et al, also found that 59.8% of adolescents received information about vaginal discharge from their parents^(15)^; this shows that the role of parents as role models, especially mothers, is very important in the process of growth and development of adolescent reproductive health^(36)^.

A person’s ethnic also influences a person’s perception of behaving to maintain and improve their health^(27)^, the culture and habits they have are generally always applied by family members and are hereditary so it can be concluded that ethnicity can influence a person’s vaginal discharge prevention behavior^(37)^. Based on this study, 24.5% came from the Batak tribe.

Based on the results of the study, 100% of female students experienced a type of physiological vaginal discharge. This is in line with research conducted by Ilankoon that the majority of participants 73.6% experienced physiological vaginal discharge and 77% agreed that 15-49 years old had normal physiology vaginal secretion^(38)^. Physiologically vaginal discharge occurs due to hormonal factors such as before or after menstruation, increased sexual desire, ovulation, and occurs during pregnancy^(39)^. Vaginal discharge is one of the reproductive health problems in adolescents that often disturbs and causes discomfort^(40)^, which if not treated properly can lead to infertility and cervical cancer which can lead to death^(29)^. Vaginal discharge is caused by two factors, namely infection due to bacteria, fungi, parasites, and non-infection due to poor preventive behavior such as not taking care of the genital area properly, stress, hormonal balance disorders and fatigue^(41)^, beside that Indonesia’s tropical climate makes it easier for fungi, viruses and bacteria to grow, which can lead to vaginal discharge^(42)^.

Knowledge can be interpreted as a conclusion from understanding that can be known by someone through objects including the senses that someone has^(43)^. Knowledge is an important domain in determining human behavior. The more knowledge a person has, the more likely he is to act appropriately. Table 2 showed that 98.1% of female students had good level of knowledge about vaginal discharge. The participants are familiar with it because they got information from the internet (58.4%), school (23.2%), and their parents (14.8%). Research by Gilliam^(34)^ and Jenkins^(33)^ state that adolescents use the internet to find or collect information about health reproduction. A study in Manado showed that 90% had good knowledge of vaginal discharge; this was because adolescents had received information about reproductive health and care through biology lessons in junior and senior high schools^(44)^. Research in the city of Padang concluded that the source of information obtained increased knowledge about vaginal discharge and prevention of vaginal discharge^(45)^. A person’s knowledge is influenced by age, education, and sources of information obtained. At the age of 16-24, young women will easily accept and understand and have a high drive to seek information about reproductive health^(46)^. Good knowledge can also be influenced by education where education can increase one’s insight or knowledge because the higher the level of education, the higher one’s interest in learning more about reproductive health^(47)^. Like the method of finding information, the results of this study indicate that 58.4% of female students seek information from the internet because it is more complete and easier to reach. Although it was found that 98.1% of the participants’ knowledge was in a good category, participants still had to be given education about the complications that occur due to vaginal discharge, because based on data in table 3 shows that only 16.1% of participants now about the complications of vaginal discharge. Many people are still ignorant and unaware of preventing vaginal discharge which can threats their health not only for recent time but also for the future, as a future mother^(28)^.

Behavior is a response or reaction and individual experience in interacting with the environment that is felt and which is not felt and which is manifested in the form of knowledge, attitudes and actions^(48)^. Research conducted by Citrawati stated that 56.8% of respondents have good behavior toward the prevention of vaginal discharge because of their knowledge. This research is also supported by Mardiana and Lubis with the results that 75% of respondents have good behavior, because adolescents have high knowledge about reproductive health and respondents know about the impact of vaginal discharge^(48)^. Another study found that 96% of respondents behaved positively toward the prevention of vaginal discharge because teenagers had good knowledge^(49)^. A person’s level of knowledge can influence the behavior of preventing vaginal discharge. The results of this study indicate that first-level nursing students at one of the private universities in Tangerang were found to have a good level of knowledge and behavior about vaginal discharge. This shows that the better the knowledge about vaginal discharge, the better the behavior of preventing it.

Table 6 shows there is relationship between level of knowledge and vaginal discharge prevention behavior *p* = 0.023. 92.3% (n=143) of the participants have good level of knowledge and have good vaginal discharge prevention behavior and only 5.8% of participants with a fair level of knowledge had fair level vaginal discharge prevention behavior.

### Study limitations

Limitations can arise during the research process, namely the limitations of communication between researchers and respondents because data collection is done through filling out online questionnaires. The researcher realized that the respondents’ time was limited in filling out the online questionnaire because of the tight schedule of lectures and non-academic activities. To overcome this, the researcher will remind respondents to fill out the questionnaire and provide a long deadline for filling out.

### Contribution to the field

The results of this study are expected to add insight and knowledge so that women, especially adolescents, can behave appropriately in preventing vaginal discharge to prevent more serious reproductive disorders.

## CONCLUSION

The conclusion of this study is that the respondents have good knowledge about current and vaginal discharge they already have good preventive behavior related to the prevention of vaginal discharge. Based on these results, education about vaginal discharge can still be provided through health seminars which are given in full by health workers. Teacher and parents are expected to remain the ones who provide information about vaginal discharge to their students and their children.
